# Differentiation Between Malignant and Benign Pulmonary Nodules by Using Automated Three-Dimensional High-Resolution Representation Learning With Fluorodeoxyglucose Positron Emission Tomography-Computed Tomography

**DOI:** 10.3389/fmed.2022.773041

**Published:** 2022-03-18

**Authors:** Yung-Chi Lai, Kuo-Chen Wu, Neng-Chuan Tseng, Yi-Jin Chen, Chao-Jen Chang, Kuo-Yang Yen, Chia-Hung Kao

**Affiliations:** ^1^Department of Nuclear Medicine, PET Center, China Medical University Hospital, Taichung, Taiwan; ^2^Graduate Institute of Biomedical Electronics and Bioinformatics, National Taiwan University, Taipei, Taiwan; ^3^Center of Augmented Intelligence in Healthcare, China Medical University Hospital, Taichung, Taiwan; ^4^Division of Nuclear Medicine, Tungs’ Taichung MetroHarbor Hospital, Taichung, Taiwan; ^5^Department of Biomedical Imaging and Radiological Science, School of Medicine, College of Medicine, China Medical University, Taichung, Taiwan; ^6^Graduate Institute of Biomedical Sciences, College of Medicine, China Medical University, Taichung, Taiwan; ^7^Department of Bioinformatics and Medical Engineering, Asia University, Taichung, Taiwan

**Keywords:** pulmonary nodules, 3D high-resolution representation learning, fluorodeoxyglucose (FDG), positron emission tomography-computed tomography (PET-CT), operating characteristic curve (AUC), artificial intelligence, deep learning

## Abstract

**Background:**

The investigation of incidental pulmonary nodules has rapidly become one of the main indications for 18F-fluorodeoxyglucose (FDG) positron emission tomography (PET), currently combined with computed tomography (PET-CT). There is also a growing trend to use artificial Intelligence for optimization and interpretation of PET-CT Images. Therefore, we proposed a novel deep learning model that aided in the automatic differentiation between malignant and benign pulmonary nodules on FDG PET-CT.

**Methods:**

In total, 112 participants with pulmonary nodules who underwent FDG PET-CT before surgery were enrolled retrospectively. We designed a novel deep learning three-dimensional (3D) high-resolution representation learning (HRRL) model for the automated classification of pulmonary nodules based on FDG PET-CT images without manual annotation by experts. For the images to be localized more precisely, we defined the territories of the lungs through a novel artificial intelligence-driven image-processing algorithm, instead of the conventional segmentation method, without the aid of an expert; this algorithm is based on deep HRRL, which is used to perform high-resolution classification. In addition, the 2D model was converted to a 3D model.

**Results:**

All pulmonary lesions were confirmed through pathological studies (79 malignant and 33 benign). We evaluated its diagnostic performance in the differentiation of malignant and benign nodules. The area under the receiver operating characteristic curve (AUC) of the deep learning model was used to indicate classification performance in an evaluation using fivefold cross-validation. The nodule-based prediction performance of the model had an AUC, sensitivity, specificity, and accuracy of 78.1, 89.9, 54.5, and 79.4%, respectively.

**Conclusion:**

Our results suggest that a deep learning algorithm using HRRL without manual annotation from experts might aid in the classification of pulmonary nodules discovered through clinical FDG PET-CT images.

## Introduction

Lung cancers usually present as either abnormal lung masses or small pulmonary nodules on chest computed tomography (CT) images and are the leading cause of cancer deaths worldwide, including in Taiwan. Several guidelines have stipulated that patients at high risk must undergo annual screening with low-dose CT scanning for pulmonary nodules to be more easily discovered (1–6). These incidentally detected pulmonary nodules could be benign or malignant (7), and differentiating between them is challenging for clinical physicians. Understanding the nature of these pulmonary nodules is crucial because it has vital implications in both therapeutic and prognostic areas. Fluorodeoxyglucose-positron emission tomography (FDG PET) has played a crucial role in the diagnosis of indeterminate pulmonary nodules with CT imaging. FDG PET detects malignancy based on the high FDG uptake, which reflects the increased glucose metabolic activity of cancer cells. Traditionally, a standardized uptake value of 2.5 has been used as a threshold to differentiate between malignant and benign nodules (8, 9). There is evidence showing that dual time point (18)F-FDG PET imaging is an important non-invasive method for the differentiation of malignant and non-malignant lesions (10–13). For instance, the sequential dual-time-point [18F]FDG PET-CT examinations may increase the sensitivity and the specificity of the PET-CT method in differential palatine tonsils diagnosis according to Pietrzak et al. (11). In addition, PET is typically used as an adjunct to CT in the evaluation of suggestive nodules (11, 14–16). However, FDG PET has several intrinsic limitations in differentiating lesions with extreme metabolic rate, leading to false positives or false negatives (17). In addition, many researchers reported that ground-glass nodules with minor metabolic activities and lower SUVmax might have a high malignancy potential (18).

Artificial intelligence (AI) algorithms based on convolutional neural networks have been increasingly applied in cross-domain image translation (19). According to previous studies, machine learning (ML) models can help in detection, differentiation from benign lesions, segmentation, staging, response assessment, and prognosis determination. More specifically, researchers have found that FDG-PET-CT metrics and radiomics features had a significant role in predicting the final diagnosis of solitary pulmonary nodules (20–22).

Conventional radiographic findings that are suggestive of benignity or malignancy include size, density, stability over time, margin appearance, wall thickness, and the presence of cavitation and calcification. According to several previous studies, uptake parameters from FDG PET have shown good diagnostic performance (accuracy between 65 and 91%) (23–28) with potential improvements coming from the characterization of uptake heterogeneity. However, a meta-analysis (29) suggested that FDG PET-CT showed insufficient sensitivity and specificity for diagnosing malignant solitary pulmonary nodules; it cannot replace the “gold standard” pathology by either resection or percutaneous biopsy. Therefore, we planned not to use traditional imaging features to differentiate benign from malignant pulmonary nodules in this study. Instead, we hoped to utilize deep learning methods that learn these features directly from data, without the need of hand-engineered feature extraction from inputs (20–22, 30).

Some of the most remarkable results of AI algorithms have been produced by systems that aid in medical image diagnoses. Several state-of-the-art AI models, such as Visual Geometry Group (VGG) and ResNet, are widely used in nuclear medical imaging (31). These algorithms take advantage of stride and pooling to downsize the feature maps, which are done prior to input from a classifier. However, the aforementioned methods result in the loss of intrinsic high-resolution information of medical images. This study aimed to use high-resolution representation learning (HRRL) as the AI algorithm to retain the high-resolution imaging features, without any stride or pooling, to reserve the size of the images (32). Therefore, we implemented automated HRRL without manual annotation by an expert as the deep learning approach to aid in the differential diagnoses of FDG PET-CT scanning for pulmonary nodules.

## Materials and Methods

### Patients

**FIGURE 1:**
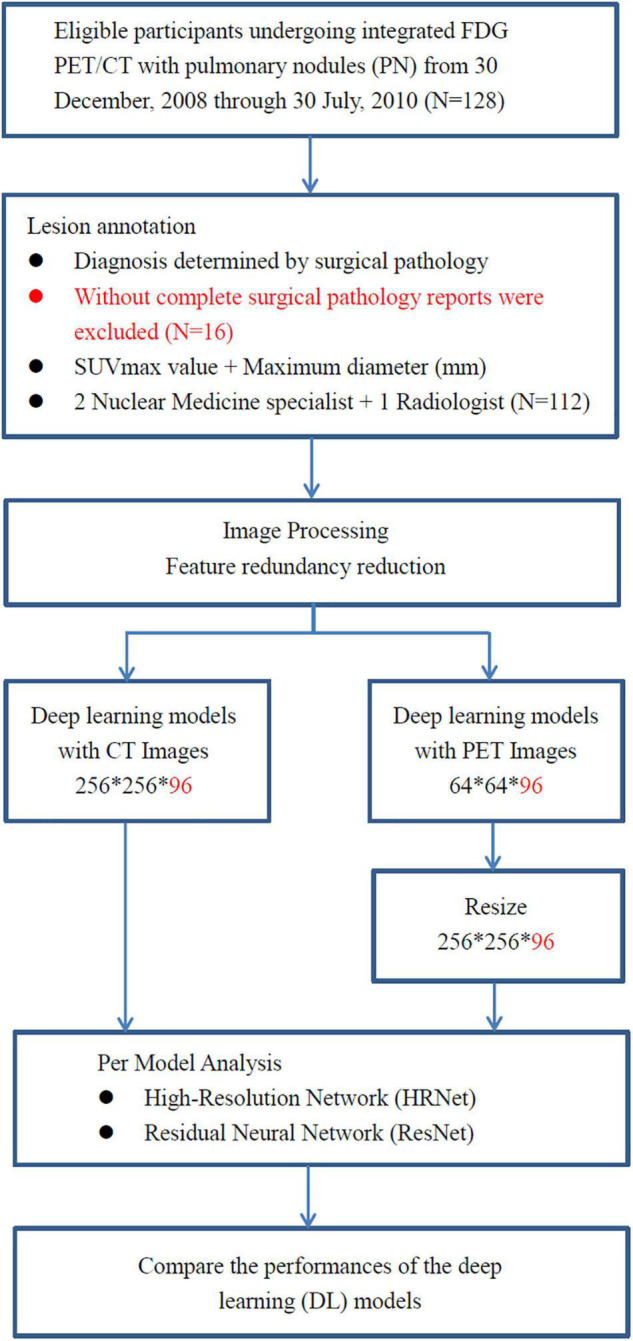
The analysis workflow of the collected dataset. A total of 112 consecutive cases of eligible patients with pulmonary nodules (PN) were enrolled in this retrospective study from 30 December 2008 through 30 July 2010 at China Medical University Hospital. Patients with pulmonary nodules had definitive diagnosis determined by surgical pathology. All of them underwent integrated FDG PET-CT prior to surgical resection of pulmonary nodules.

A total of 112 consecutive cases of eligible patients (age range 29–85 years; mean age 62.28) with pulmonary nodules (PN) were enrolled in this retrospective study from 30 December 2008 through 30 July 2010 at China Medical University Hospital. Patients were selected for this study according to the following criteria: (a) underwent integrated FDG PET-CT and (b) had definitive diagnosis determined by surgical pathology (Figure 1). The final study group of 112 patients comprised 60 men and 52 women. Overall, pulmonary nodules detected by CT of the chest were divided into two groups (i.e., benign and malignant) as diagnosed by surgical pathology (Table 1). The first group comprised 33 benign nodules (mean diameter: 24.88 ± 16.49 mm), 4 of them were ground-glass nodules (GGN) and the other 29 were solid nodules. The second group comprised 79 malignant nodules (mean diameter: 29.86 ± 18.99 mm), 12 of them were GGN and the other 67 were solid nodules. The imaging and clinical data of these patients were reviewed and analyzed retrospectively. This study was approved by the Ethics Committee of our hospital [DMR99-IRB-010-(CR-12)].

**TABLE 1:** Patient characteristics.

Patient	Benign	Malignant	*P*-value
**Sex**			
Male	21	39	0.120
Female	12	40	
**Age**			
Age, mean (years)^b^	58.55 ± 13.31	63.84 ± 11.60	0.038*
Age, range (years)	29–85	39–82	
**Pathology of pulmonary nodules (PNs)**			
Diameter, means (mm)^b^	24.88 ± 16.49	29.86 ± 18.99	0.192
Early maximum SUV^b^	2.97 ± 3.30	5.20 ± 3.80	0.004*
Delayed maximum SUV^b^	3.45 ± 3.82	6.13 ± 4.56	0.002*
Solid/GGN^a^	29/4	67/12	0.775
Total	33 (29.46)	79 (70.54)	

*PNs, pulmonary nodules; Excluded, without surgical pathology reports; GGN, ground-glass nodules.*

*^a^Chi-square (χ^2^) test.*

*^b^Student’s t-test.*

*The p-value of <0.05 was considered statistically.

### Fluorodeoxyglucose Positron Emission Tomography-Computed Tomography Imaging Protocol

All patients were asked to fast for at least 4 h before FDG PET-CT imaging. Imaging was performed with a PET-CT scanner (Discovery STE, GE Medical Systems, Milwaukee, WI, United States). Whole-body FDG PET-CT images were acquired approximately 45 min after intravenous injection of 370 MBq (10 mCi) of FDG. Delayed FDG PET-CT images were obtained approximately 70 min after FDG injection (33–35). In this study, however, we only adopted the delayed FDG PET-CT images for further preprocessing and input to the deep learning models. PET emission images were acquired after CT scans at 2 min per field of view in the three-dimensional acquisition mode. The CT images were reconstructed onto a 512 × 512 matrix with a section thickness of 3.75 mm, reconstructed onto a 128 × 128 matrix, and converted into 511 keV equivalent attenuation factors for attenuation correction of the corresponding PET emission images. The maximum SUVmax of lung nodules on early and delayed FDG PET-CT images were measured.

### Preprocessing for Automated Models

**FIGURE 2:**
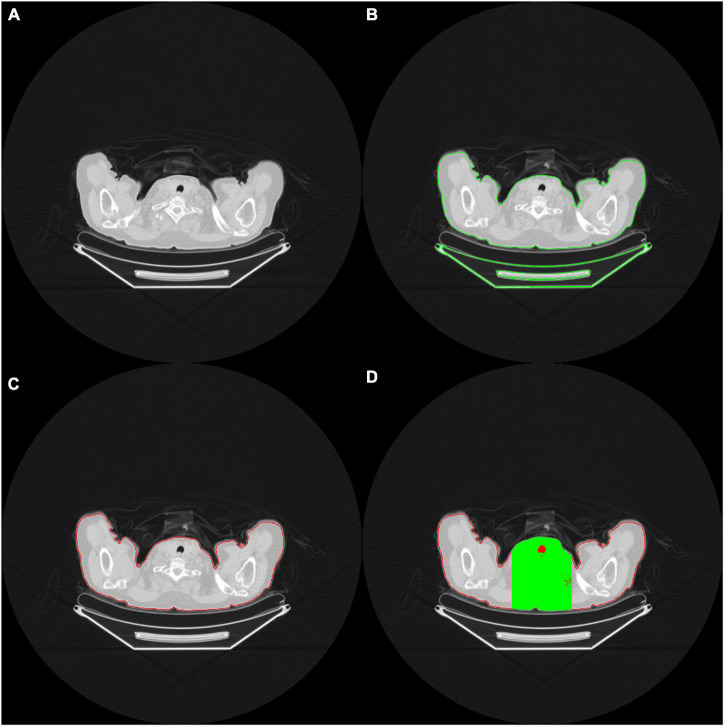
**(A)** The red contour delineates the air (black area) within the tracheal lumen, which helps the program determine the upper edge of the lungs **(B)** OpenCV identifies the contour area of the body on the CT images **(C)** Body contour block (red contour block) **(D)** Green area indicates the limited calculation area. Only the black area in the green range is included in the calculation of the ratio.

We defined each patient’s lung territories by using the mediastinal window on the CT images. The CT mediastinal window level (WL) was 40, and the window width (WW) was 400. Therefore, values less than −160 were rendered entirely black, and values >240 were rendered entirely white (Figure 2A). Under this setting, the tracheal lumen and lung parenchyma appeared to be almost black. Such an image preprocessing can help the program automatically determine which trans-axial slice of image is the upper edge of the lung parenchyma. The lungs were indicated by the presence of air.

In order to accurately determine the contour of bilateral lung regions, the body block must be found first. This study used the Python Open Source Computer Vision Library (OpenCV) package. It comes free with a cross-platform program and can execute a few functions, such as finding contours. The contour function can perform threshold calculations for grayscale images or customize the threshold of the cutting block to optimize the contour finding function. In this study, we used the contour function of the OpenCV package to identify all the contours on the CT images (Figure 2B).

When identifying the contour block of CT images in OpenCV, determining the contour block of the body is essential. The contour area of the body can be determined according to the center of gravity and the size of the contour area (Figure 2C). A contour area with its center of gravity biased toward the edge area or with an overly small size is generally not the main body area.

After identifying the body contour area, we determined the upper edge of the lung parenchyma within the body area. As we viewed the consecutive trans-axial slices of the CT images from the top to the button, the lung parenchyma usually begins at the level where the air (i.e., black area) does. When the block area of the uppermost lung air exceeds a certain percentage (e.g., 5%) of the body block area, the block is adopted as the starting level of the lungs. However, in this circumstance, the presence of some unusual, poor quality CT images results in the presence of many hollow black areas in the body contour block, which may cause errors in the capture. Therefore, to determine the area of the body block accurately, we took the center of the images as the starting point and extended to the left and right of it until the framed area was 33% of the body block area. The derived region (marked in green on Figure 2D) was then defined as the calculation region of interest, and only the black area (i.e., air) within that region (marked in red on Figure 2D) was subsequently adopted for further calculation. When the proportion of air exceeded a particular proportion (e.g., 5% of the body block area), the trans-axial slice of that image was regarded as depicting the upper edge of the lung parenchyma, and the image was ready to be captured for training.

To obtain accurate three-dimensional (3D) CT images and to improve training efficiency, the body contour area was obtained from the determined uppermost level trans-axial image slice of the lung parenchyma. The center of gravity of the body contour area was identified. Subsequently, we retrieved 256-pixel wide images that extended outward based on the center of gravity of the body contour block, followed by obtaining the counterpart PET images. From the uppermost level trans-axial image slice of the lung parenchyma, 96 consecutive trans-axial image slices were captured downward from both CT and PET images.

**FIGURE 3:**
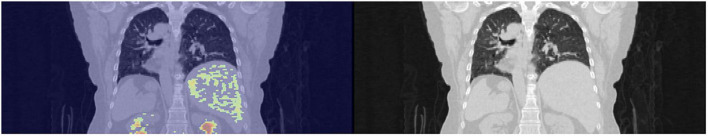
Extraction of the 3D images of the lungs with a thickness of 96 slices of trans-axial CT images.

The 96 images retrieved from CT and PET, respectively, were of the same thickness and size and could cover the entire bilateral lung regions. Finally, both CT and PET 3D images from 112 patients were obtained for subsequent input to the deep learning models. The 3D image data were unified into 256 × 256 × 96 and 64 × 64 × 96 for the CT and PET images, respectively (Figure 3).

### Preprocessing by Manual Annotation

An experienced nuclear physician carried out conventional manual annotation by determining the representative image slices that contain the maximum diameters (i.e., tumor coordinates) of pulmonary nodules. A total of 16 consecutive image slices (adjacent to the aforementioned representative image slices) were then retrieved. We cropped out CT images of 64 × 64 × 16 and PET images of 16 × 16 × 16, followed by resizing the PET images to the same size as the CT images.

### Input to Deep Learning Models

Under the lung window setting (WL: −400 and WW: 1,500), we normalized the input image data by converting the data to a value from 0 to 1. For PET images, the maximum value in the image range was normalized. Furthermore, PET image data were converted into values ranging from 0 to 1 to enhance the convergence efficiency of the model.

**FIGURE 4:**
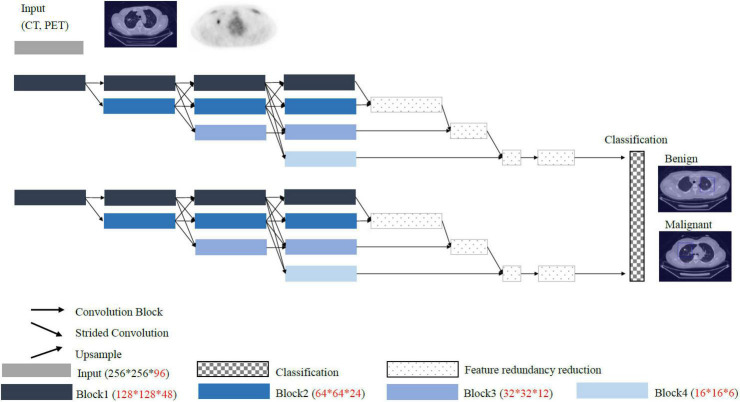
Overall structure of the proposed deep learning model (i.e., HRNet).

Conventional convolutional neural network such as VGG and ResNet models pass through the stride and pooling layers to continuously reduce the sizes of the feature maps and finally enter the classifiers (Figure 4). However, reducing the sizes of the features leads to the loss of resolution. Therefore, we proposed a High-Resolution Network (HRNet) architecture with a view to preserving high-resolution features. The top layer of HRNet does not pass through any of the stride and pooling layers so that the features were able to retain their sizes.

### Statistical Analyses

**FIGURE 5:**
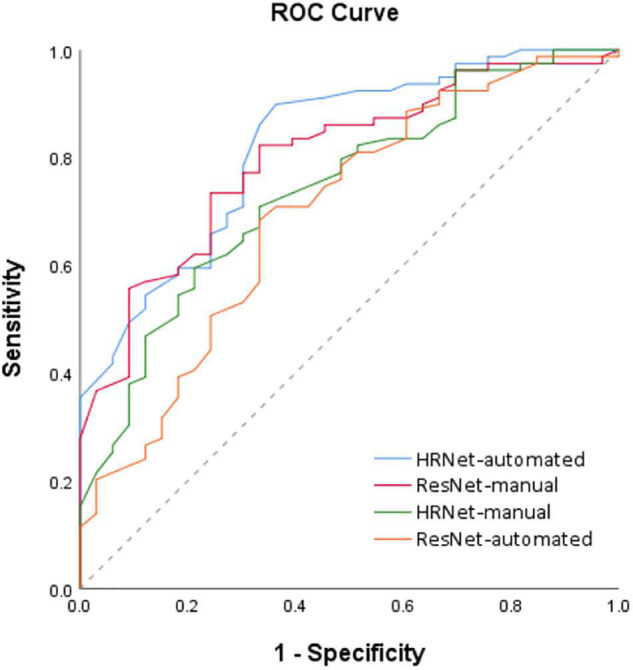
ROC analysis of the 4 models based on the PET-CT images. Receiver operating characteristic (ROC) curves for the four deep learning models (i.e., HRNet-automated, HRNet-manual, ResNet- automated and ResNet-manual) based on the PET-CT images. The area under the ROC curve (AUC) of the HRNet-automated, HRNet-manual, ResNet-automated, and ResNet-manual models were 0.781 (95% CI = 0.755–0.834), 0.789 (95% CI = 0.761–0.906), 0.652 (95% CI = 0.582–0.737), and 0.743 (95% CI = 0.680–0.842), respectively.

The statistical analysis was performed using SPSS 26 statistical software (IBM, Armonk, NY, United States) and MedCalc statistical software^1^. Fisher’s exact or Chi-square (χ^2^) test was used to compare categorical variables. Student’s *t*-test was used to compare continuous variables between groups as appropriate. The *p*-value of <0.05 was considered statistically significant. MedCalc statistical software was used to perform ROC curve analysis, the value of the models was predicted based on the ROC curve analysis and corresponding 95% confidence intervals (CIs) were calculated. ROC analysis for the models prediction with benign and malignant lesion revealed an area under the ROC curve (AUC) of 0.781 (95% CI = 0.755–0.834), 0.789 (95% CI = 0.761–0.906), 0.652 (95% CI = 0.582–0.737), and 0.743 (95% CI = 0.680–0.842) for High-Resolution Network (HRNet)-automated, High-Resolution Network (HRNet)-manual, Residual Network (ResNet)-automated, and Residual Network (ResNet)-manual models, respectively (Figure 5).

**FIGURE 6:**
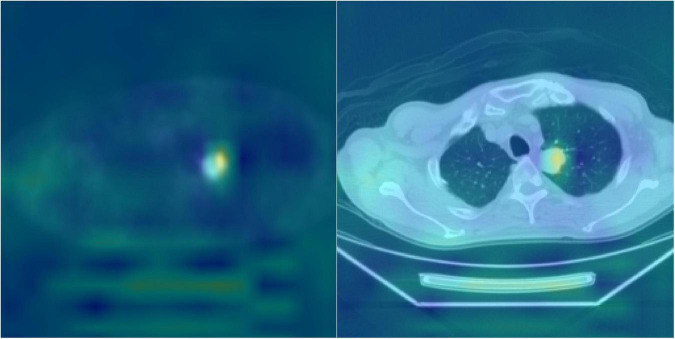
Heatmap visualization of the HRNet-automated model. The highlighted (e.g., red) area on the heat map were used to indicate the portions/pixels of an image that have the greatest contribution to the output of the model. The heated area matched quite well with the actual locations of pulmonary nodules on the DICOM PET-CT images and the clinical records, which potentially serves as a beneficial clinical tool for patient treatment planning.

Deep learning often uses heat maps to differentiate regions with characteristic features (Figure 6). The lungs are adjacent to other internal organs and tissues, such as the heart, liver, and lymph nodes. If these organs also exhibit high FDG uptake, they may disrupt the focus of the deep learning model and affect the accuracy (ACC) of the analysis. Therefore, the heat map is based on the feature map in the last layer of the proposed model and is concentrated on the area of pulmonary nodules.

## Results

The effects of the two groups of PET-CT images on the differentiation between benign and malignant pulmonary nodules were evaluated. First, when applied only to automatically detected pulmonary nodules on the PET-CT images, the HRNet model [sensitivity (SEN): 0.899; specificity (SPE): 0.545; ACC: 0.795] performed significantly better than the ResNet model (SEN: 0.785; SPE: 0.394, ACC: 0.670) did. Compared with the ResNet model, the HRNet performed better in terms of SEN and ACC. HRNet and ResNet models performed comparably when applied only to the PET-CT images of pulmonary nodules. The ACC of the ResNet model was only slightly better than that of the HRNet model. In summary, HRNet is suitable for two types of PET-CT datasets, and its performance is more stable than that of ResNet.

### Model Comparison

Compared with the traditional model, the HRNet-automated model used in this study performed significantly better than the ResNet-automated models. When models which automatically detected pulmonary nodules were compared with each other, HRNet performed significantly better than ResNet (significance level: 0.0036) did. The performance for the HRNet-automated versus that for the ResNet-manual (significance level: 0.3343) did not significantly differ, indicating that this study’s method, which functions without manual expert annotation, yield comparable predictions as traditional labeling models that require expert annotation (Table 2).

**TABLE 2:** Model comparison.

Model comparison	Significance level, p*
HRNet-automated vs. HRNet-manual	0.6526
HRNet-automated vs. ResNet-automated	0.0036*
HRNet-automated vs. ResNet-manual	0.3343
HRNet-manual vs. ResNet-automated	0.0039*
HRNet-manual vs. ResNet-manual	0.6398
ResNet-automated vs. ResNet-manual	0.0014*
ResNet-PET-CT vs. ResNet-CT	0.0749
HRNet-PET-CT vs. HRNet-CT	0.7422

*The p-value of <0.05 was considered statistically.

The results of manual and automated detection show that the prediction performance of ResNet is low in automated detection (AUC = 0.652), and the prediction effect must be improved by manually labeling detection (AUC = 0.743). The results of HRNet manual (AUC = 0.789) and automated detection (AUC = 0.781) have comparable prediction performances.

According to the ROC curves based on image data from automated detection, the prediction performance of HRNet using PET-CT (AUC = 0.781) is moderately better than that of only using CT (AUC = 0.725); and the performance of ResNet utilizing PET-CT (AUC = 0.652) is also better than that of utilizing only CT (AUC = 0.566).

The sensitivity (0.899) and AUC of HRNet-PET-CT-automated are moderately better than those of HRNet-CT-automated (*p*-value = 0.742). Although the specificity of HRNet-PET-CT-automated (0.545) is slightly lower than that of HRNet-CT-automated, it does not markedly affect the overall prediction performance, as evidenced by the satisfactory precision (0.795) of HRNet-PET-CT-automated. Therefore, the HRNet-PET-CT-automated model is quite effective in predicting malignant pulmonary nodules, which is also one of the most prominent findings in this research.

## Discussion

With the advancement of chest CT and plain radiographs, the detection rate of pulmonary nodules has drastically improved (36, 37). The assessment of pulmonary nodules is crucial because they may be the first indications of early lung neoplasm. Approximately 35% of single pulmonary nodules are primary malignancies (38). Differentiating small nodules into malignant and benign nodules is challenging because of their small size and lack of specific morphological features (39). A study reported that approximately half of lung cancer patients missed the optimal time for surgical treatment, resulting in a decrease in the 5-year survival rate by 10–15% (40). Therefore, the accurate diagnosis of patients with pulmonary nodules helps to improve the ACC of the initial cancer staging and prognosis of patients with malignant nodules (23).

Nevertheless, pulmonary nodules are not all malignant lesions. Pulmonary nodules, except in lung cancer, can be inflammatory or infectious lesions and can have other relatively rare benign etiologies. The prevalence of lung cancer in pulmonary nodules is high, and the early detection of malignant nodules might improve the chances of successful treatment. Transbronchial needle aspiration biopsy, percutaneous transthoracic biopsy, or video-assisted thoracoscopic surgery can yield histopathological information. However, they have variable ACC in diagnosing lung cancer because these are invasive procedures dependent on the diameter and position of the nodule and whose execution is dependent on skill.

FDG-PET-CT is commonly used in the diagnosis of pulmonary nodules. It can be used to detect malignancy through high FDG accumulation, which indicates the presence of metabolically active cancer cells by quantifying the rate of cellular glucose metabolism. Malignant nodules generally have increased glucose transporter expression and metabolic activity, which is evidenced by a high FDG uptake (41). However, benign lesions also occasionally exhibit increased metabolic activity, such as infections, tuberculosis, and granulomatous disease (29, 42–44). Conversely, malignant lung lesions with false-negative findings on PET scan may be relevant tumors with low glycolytic activity (such as adenocarcinomas, bronchioloalveolar carcinomas, carcinoid tumors, and low-grade lymphomas), small-sized tumors (partial volume effect), or metastasized tumors with a mucinous component (relative low cellularity) (45). In pooled results from a meta-analysis, FDG-PET-CT had a diagnostic SEN of 0.89 [95% confidence interval (CI), 0.87–0.91] and an SPE of 0.70 (95% CI, 0.66–0.73) in the diagnosis of malignant solitary pulmonary nodules (29). Thus, at present, the evidence in the literature indicates only moderate ACC for FDG-PET-CT in differentiating malignant from benign pulmonary nodules. Further research is required to improve its reliability.

Recently, several studies have applied deep learning or machine learning approaches to conventional CT and FDG-PET-CT images to diagnose lung cancer (20–22). A study evaluated the performance of a deep learning system for the differential diagnosis of lung cancer with conventional CT and FDG-PET-CT by using transfer learning and metadata (46). The introduction of metadata and data on SUVmax and lesion size derived from PET-CT into baseline CT models improved diagnostic performance for models applied to CT images derived from PET-CT [area under the receiver operating characteristic curve (AUC) = 0.837 vs. 0.762] or conventional CT images (AUC = 0.877 vs. 0.817) models.

In our study, we sought to assess whether deep learning may be helpful in the differentiation of pulmonary nodules in FDG PET-CT imaging. In total, 112 patients with pulmonary nodules who underwent FDG PET-CT before surgery were enrolled retrospectively. The images of the lungs were automatedly extracted through deep HRRL without the aid of an expert. The deep convolutional networks were trained within a HRNet framework, which executes high-resolution classification instead of using the conventional segmentation method to provide more precise localization of image data.

The performance of two-dimensional (2D) and 3D networks were evaluated. According to a recently published study, the additional spatial dimension of the 3D network substantially improved the quality of the inference because the additional dimension allowed an equivalent 3D network to produce volumes with higher fidelity across all spatial dimensions. Therefore, we implemented the 3D model instead of the conventional 2D model. The lung images obtained were then fed to the HRRL algorithm to automatically classify the detected pulmonary nodules into malignant and benign ones, followed by an assessment of ACC. To our knowledge, our study is the first to determine the value of deep learning for the automated classification of pulmonary nodules on FDG-PET-CT images.

Conventional deep learning model architectures generally use a classification task model as the primary framework. When the image is being extended, its size is gradually compressed, which compromises its high-resolution representations. Therefore, we applied the conventional deep learning algorithm for the overall 3D image classification, which led to decreased diagnostic ACC and loss of features.

The proposed HRNet retains the high-resolution features. However, it is challenging to solve multiscale problems effectively by using only high-resolution representations. Therefore, we implement the proposed architecture to preserve high-resolution features. The network solves multiscale problems through continuous reduction, followed by multiscale fusions, to maintain the resolution of various scales. Therefore, in this study, HRNet used for lung image recognition did not lead to the loss of pulmonary nodule characteristics (47, 48).

With regard to the overall 3D image classification target processed in this study, the aforementioned reduced size feature causes the loss of regional lung features and decreases ACC.

High-Resolution Network maintains high-resolution features, but it is not easy to extract deep textural features using only high-resolution features. Therefore, the proposed architecture preserves high-resolution features. It aims to solve the multiscale problem through continuous shrinking. The architecture of HRNet is similar to that of ICLR’18-MSDNet, which works through multiscale fusion and preserves high resolution.

The main architecture of HRNet acts to integrate each branch of the feature map. The resolution of each feature map is different, and the actual operation involves the use of upsampling and downsampling to achieve integration. Although the resolution of each branch is different, the features are fused through the multiscale fusion method to extract meaningful information for overall 3D image classification.

Furthermore, we used 3D HRRL and compared the ACC values of two types of model input: The CT component of PET-CT input, and the combined PET-CT input based on FDG PET-CT imaging. Although most previous studies assessing the ACC of AI algorithm in differentiating malignant from benign lesions have taken advantage of a combination of different tests as the reference standard, such as clinical follow-up with additional imaging for some study participants and histopathology for others, surgical pathology was the sole definitive reference standard used for all individuals in this study.

The significant findings of our study are detailed as follows: First, deep learning might be a promising technique for the detection and differentiation of pulmonary nodules on FDG PET-CT images. Although our study only consisted of 112 patients, the AI algorithm generally gave accurate and reliable results. Second, the lung images could be extracted automatically through HRRL in the absence of any manual delineation. Third, the deep HRNet kept the high resolution of the images intact, unlike in other well-known AI models, such as VGG and ResNet, which compromised their resolution at each round of k-fold cross-validation (46). Fourth, the performance metrics of the combined PET-CT model were generally better than those of the model derived from the CT component solely.

## Conclusion

This retrospective study indicates that automated 3D HRRL with FDG-PET-CT has promising performance in distinguishing between malignant and benign pulmonary nodules. One of the most significant strengths of the proposed deep learning algorithm is that it can potentially automatically detect and classify pulmonary nodules without any time-consuming manual annotation. However, this study had a limited number of participants, and an extensive multicenter study with external validation is required for further verification of the results.

## Data Availability Statement

The original contributions presented in the study are included in the article/supplementary material, further inquiries can be directed to the corresponding author/s.

## Ethics Statement

This study was approved by the Ethics Committee of our hospital [DMR99-IRB-010-(CR-11)]. Written informed consent for participation was not required for this study in accordance with the National Legislation and the Institutional Requirements.

## Author Contributions

Y-CL, K-CW, and C-HK: conception and design. C-HK: administrative support. All authors: significant contributions, agreement with the content of the manuscript, collection and assembly of data, data analysis and interpretation, manuscript writing, and final approval of manuscript.

## Conflict of Interest

The authors declare that the research was conducted in the absence of any commercial or financial relationships that could be construed as a potential conflict of interest.

## Publisher’s Note

All claims expressed in this article are solely those of the authors and do not necessarily represent those of their affiliated organizations, or those of the publisher, the editors and the reviewers. Any product that may be evaluated in this article, or claim that may be made by its manufacturer, is not guaranteed or endorsed by the publisher.

## Footnotes

1
www.medcalc.org

## References

[1] Moyer VA U.S. Preventive Services Task Force. Screening for lung cancer: U.S. preventive services task torce recommendation statement. *Ann Intern Med.* (2014) 160:330–8. 24378917 10.7326/M13-2771

[2] Rivera MP Mehta AC Wahidi MM. Establishing the diagnosis of lung cancer: diagnosis and management of lung cancer, 3rd ed: American college of chest physicians evidence-based clinical practice guidelines. *Chest.* (2013) 143:e142S–e165S. 10.1378/chest.12-2353 23649436

[3] Detterbeck FC Mazzone PJ Naidich DP Bach PB. Screening for lung cancer: diagnosis and management of lung cancer, 3rd ed: American college of chest physicians evidence-based clinical practice guidelines. *Chest.* (2013) 143:e78S–e92S. 10.1378/chest.12-2350 23649455 PMC3749713

[4] Wender R Fontham ET Barrera E Jr. Colditz GA Church TR Ettinger DS American cancer society lung cancer screening guidelines. *CA Cancer J Clin.* (2013) 63:107–17. 23315954 10.3322/caac.21172 PMC3632634

[5] Roberts H Walker-Dilks C Sivjee K Ung Y Yasufuku K Hey A Screening high-risk populations for lung cancer: guideline recommendations. *J Thorac Oncol.* (2013) 8:1232–7. 10.1097/JTO.0b013e31829fd3d5 24457233

[6] Jacobson FL Austin JH Field JK Jett JR Keshavjee S MacMahon H Development of the american association for thoracic surgery guidelines for low-dose computed tomography scans to screen for lung cancer in North America: recommendations of the American association for thoracic surgery task force for lung cancer screening and surveillance. *J Thorac Cardiovasc Surg.* (2012) 144:25–32. 10.1016/j.jtcvs.2012.05.059 22710038

[7] Wahidi MM Govert JA Goudar RK Gould MK McCrory DC American College of Chest Physicians. Evidence for the treatment of patients with pulmonary nodules: when is it lung cancer?: ACCP evidence-based clinical practice guidelines (2nd edition). *Chest.* (2007) 132:94S–107S. 10.1378/chest.07-1352 17873163

[8] Patz EF Jr. Lowe VJ Hoffman JM Paine SS Burrowes P Coleman RE Focal pulmonary abnormalities: evaluation with F-18 fluorodeoxyglucose PET scanning. *Radiology.* (1993) 188:487–90. 10.1148/radiology.188.2.8327702 8327702

[9] Knight SB Delbeke D Stewart JR Sandler MP. Evaluation of pulmonary lesions with FDG-PET. Comparison of findings in patients with and without a history of prior malignancy. *Chest.* (1996) 109:982–8. 10.1378/chest.109.4.982 8635381

[10] Houshmand S Salavati A Segtnan EA Grupe P Høilund-Carlsen PF Alavi A. Dual-time-point imaging and delayed-time-point fluorodeoxyglucose-PET/computed tomography imaging in various clinical settings. *PET Clin.* (2016) 11:65–84. 10.1016/j.cpet.2015.07.003 26590445

[11] Pietrzak AK Kazmierska J Marszalek A Cholewinski W. Evaluation of physiologic and abnormal glucose uptake in palatine tonsils: differential diagnostics with sequential dual-time-point 2-deoxy-2-[18F]FDG PET/CT. *Q J Nucl Med Mol Imaging.* (2020) 64:299–306. 10.23736/S1824-4785.18.03065-0 30221906

[12] Shimizu K Okita R Saisho S Yukawa T Maeda A Nojima Y Clinical significance of dual-time-point 18F-FDG PET imaging in resectable non-small cell lung cancer. *Ann Nucl Med.* (2015) 29:854–60. 10.1007/s12149-015-1013-3 26254228 PMC4666280

[13] Pietrzak A Marszalek A Paterska M Golusinski P Narozna J Cholewinski W. Initial and delayed metabolic activity of palatine tonsils measured with the PET/CT-dedicated arameters. *Diagnostics (Basel).* (2020) 10:836. 10.3390/diagnostics10100836 33080852 PMC7603072

[14] Sanz-Viedma S Torigian DA Parsons M Basu S Alavi A. Potential clinical utility of dual time point FDG-PET for distinguishing benign from malignant lesions: implications for oncological imaging. *Rev Esp Med Nucl.* (2009) 28:159–66. 10.1016/s1578-200x(09)90000-6 19558958

[15] Koppenol WH Bounds PL Dang CV. Otto Warburg’s contributions to current concepts of cancer metabolism. *Nat Rev Cancer.* (2011) 11:325–37. 10.1038/nrc3038 21508971

[16] Beyer T Townsend DW Brun T Kinahan PE Charron M Roddy R A combined PET/CT scanner for clinical oncology. *J Nucl Med.* (2000) 41:1369–79. 10945530

[17] Pauwels EK Ribeiro MJ Stoot JH McCready VR Bourguignon M Mazière B. FDG accumulation and tumor biology. *Nucl Med Biol.* (1998) 25:317–22. 10.1016/s0969-8051(97)00226-6 9639291

[18] Goudarzi B Jacene HA Wahl RL. Diagnosis and differentiation of bronchioloalveolar carcinoma from adenocarcinoma with bronchioloalveolar components with metabolic and anatomic characteristics using PET/CT. *J Nucl Med.* (2008) 49:1585–92. 10.2967/jnumed.108.052712 18794276

[19] Schaefferkoetter J Yan J Moon S Chan R Ortega C Metser U Deep learning for whole-body medical image generation. *Eur J Nucl Med Mol Imaging.* (2021) 48:3817–26. 10.1007/s00259-021-05413-0 34021779

[20] Zaharchuk G Davidzon G. Artificial Intelligence for optimization and interpretation of PET/CT and PET/MR images. *Semin Nucl Med.* (2021) 51:134–42. 10.1053/j.semnuclmed.2020.10.001 33509370

[21] Sadaghiani MS Rowe SP Sheikhbahaei S. Applications of artificial intelligence in oncologic 18F-FDG PET/CT imaging: a systematic review. *Ann Transl Med.* (2021) 9:823. 10.21037/atm-20-6162 34268436 PMC8246218

[22] Albano D Gatta R Marini M Rodella C Camoni L Dondi F Role of 18F-FDG PET/CT radiomics features in the differential diagnosis of solitary pulmonary nodules: diagnostic accuracy and comparison between two different PET/CT scanners. *J Clin Med.* (2021) 10:5064. 10.3390/jcm10215064 34768584 PMC8584460

[23] Tang K Wang L Lin J Zheng X Wu Y. The value of 18F-FDG PET/CT in the diagnosis of different size of solitary pulmonary nodules. *Medicine (Baltimore).* (2019) 98:e14813. 10.1097/MD.0000000000014813 30882661 PMC6426628

[24] Evangelista L Cuocolo A Pace L Mansi L Vecchio SD Miletto P Performance of FDG-PET/CT in solitary pulmonary nodule based on pre-test likelihood of malignancy: results from the Italian retrospective multicenter trial. *Eur J Nucl Med Mol Imaging.* (2018) 45:1898–907. 10.1007/s00259-018-4016-1 29736699

[25] Wang L Chen Y Tang K Lin J Zhang H. The value of 18F-FDG PET/CT mathematical prediction model in diagnosis of solitary pulmonary nodules. *Biomed Res Int.* (2018) 2018:9453967. 10.1155/2018/9453967 29789808 PMC5896270

[26] Chen S Harmon S Perk T Li X Chen M Li Y Using neighborhood gray tone difference matrix texture features on dual time point PET/CT images to differentiate malignant from benign FDG-avid solitary pulmonary nodules. *Cancer Imaging.* (2019) 19:56. 10.1186/s40644-019-0243-3 31420006 PMC6697997

[27] Taralli S Scolozzi V Foti M Ricciardi S Forcione AR Cardillo G 18F-FDG PET/CT diagnostic performance in solitary and multiple pulmonary nodules detected in patients with previous cancer history: reports of 182 nodules. *Eur J Nucl Med Mol Imaging.* (2019) 46:429–36. 10.1007/s00259-018-4226-6 30535767

[28] Karyagar S Koç Z Karyagar S Bekar Y. Diagnostic performance of 18F-FDG PET/CT in solitary pulmonary nodules of non-smokers. *Turk J Thorac Cardiovasc Surg.* (2017) 25:235–41.

[29] Li ZZ Huang YL Song HJ Wang YJ Huang Y. The value of 18F-FDG-PET/CT in the diagnosis of solitary pulmonary nodules: a meta-analysis. *Medicine (Baltimore).* (2018) 97:e0130. 10.1097/MD.0000000000010130 29561412 PMC5895332

[30] Chartrand G Cheng PM Vorontsov E Drozdzal M Turcotte S Pal CJ Deep learning: a primer for radiologists. *Radiographics.* (2017) 37:2113–31. 10.1148/rg.2017170077 29131760

[31] Puttagunta M Ravi S. Medical image analysis based on deep learning approach. *Multimed Tools Appl.* (2021) 80:24365–98. 10.1007/s11042-021-10707-4 33841033 PMC8023554

[32] Wang J Sun K Cheng T Jiang B Deng C Zhao Y Deep high-resolution representation learning for visual recognition. *IEEE Trans Pattern Anal Mach Intell.* (2020) 43:3349–64. 10.1109/TPAMI.2020.2983686 32248092

[33] Conrad GR Sinha P. Narrow time-window dual-point 18F-FDG PET for the diagnosis of thoracic malignancy. *Nucl Med Commun.* (2003) 24:1129–37. 10.1097/00006231-200311000-00002 14569166

[34] Schillaci O Travascio L Bolacchi F Calabria F Bruni C Cicciò C Accuracy of early and delayed FDG PET-CT and of contrast-enhanced CT in the evaluation of lung nodules: a preliminary study on 30 patients. *Radiol Med.* (2009) 114:890–906. 10.1007/s11547-009-0400-z 19579015

[35] Chen YM Huang G Sun XG Liu JJ Chen T Shi YP Optimizing delayed scan time for FDG PET: comparison of the early and late delayed scan. *Nucl Med Commun.* (2008) 29:425–30. 10.1097/MNM.0b013e3282f4d389 18391725

[36] Leef JL III Klein JS. The solitary pulmonary nodule. *Radiol Clin North Am.* (2002) 40:123–43. 11813815 10.1016/s0033-8389(03)00113-1

[37] Seemann MD Seemann O Luboldt W Bonél H Sittek H Dienemann H Differentiation of malignant from benign solitary pulmonary lesions using chest radiography, spiral CT and HRCT. *Lung Cancer.* (2000) 29:105–24. 10.1016/s0169-5002(00)00104-5 10963841

[38] Jemal A Thomas A Murray T Thun M. Cancer statistics, 2002. *CA Cancer J Clin.* (2002) 52:23–47. 11814064 10.3322/canjclin.52.1.23

[39] Swensen SJ. CT screening for lung cancer. *AJR Am J Roentgenol.* (2002) 179:833–6. 12239020 10.2214/ajr.179.4.1790833

[40] Fletcher JW Kymes SM Gould M Alazraki N Coleman RE Lowe VJ A comparison of the diagnostic accuracy of 18F-FDG PET and CT in the characterization of solitary pulmonary nodules. *J Nucl Med.* (2008) 49:179–85. 10.2967/jnumed.107.044990 18199626

[41] Mochizuki T Tsukamoto E Kuge Y Kanegae K Zhao S Hikosaka K FDG uptake and glucose transporter subtype expressions in experimental tumor and inflammation models. *J Nucl Med.* (2001) 42:1551–5. 11585872

[42] Ost D Fein AM Feinsilver SH. Clinical practice. The solitary pulmonary nodule. *N Eng J Med.* (2003) 348:2535–42. 10.1056/NEJMcp012290 12815140

[43] Chen CJ Lee BF Yao WJ Cheng L Wu PS Chu CL Dual-phase 18F-FDG PET in the diagnosis of pulmonary nodules with an initial standard uptake value less than 2.5. *AJR Am J Roentgenol.* (2008) 191:475–9. 10.2214/AJR.07.3457 18647920

[44] Huang YE Lu HI Liu FY Huang YJ Lin MC Chen CF Solitary pulmonary nodules differentiated by dynamic F-18 FDG PET in a region with high prevalence of granulomatous disease. *J Radiat Res.* (2012) 53:306–12. 10.1269/jrr.11089 22374400

[45] Chang JM Lee HJ Goo JM Lee HY Lee JJ Chung JK False positive and false negative FDG-PET scans in various thoracic diseases. *Korean J Radiol.* (2006) 7:57–69. 10.3348/kjr.2006.7.1.57 16549957 PMC2667579

[46] Park YJ Choi D Choi JY Hyun SH. Performance evaluation of a deep learning system for differential diagnosis of lung cancer with conventional CT and FDG PET/CT using transfer learning and metadata. *Clin Nucl Med.* (2021) 46:635–40. 10.1097/RLU.0000000000003661 33883488

[47] He K Zhang X Ren S Sun J. Deep residual learning for image recognition. *arXiv.* (2015) [Prerint]. 1512.03385 [cs.CV].

[48] Sun K Xiao B Liu D Wang J. Deep high-resolution representation learning for human pose estimation. *arXiv.* (2019) [Prerint]. 1902.09212 [cs.CV].

